# Appropriately Reduced Nitrogen and Increased Phosphorus in Ratooning Rice Increased the Yield and Reduced the Greenhouse Gas Emissions in Southeast China

**DOI:** 10.3390/plants13030438

**Published:** 2024-02-02

**Authors:** Yuncheng Yang, Feifei Yao, Yangbo Sun, Zhipeng Yang, Rong Li, Ge Bai, Wenxiong Lin, Hongfei Chen

**Affiliations:** 1 College of JunCao Sciences and Ecology, Fujian Agriculture and Forestry University, Fuzhou 350002, China; 15899062591@163.com (Y.Y.); 17872351005@163.com (F.Y.); s15615538017@163.com (Y.S.); m18350032268@163.com (Z.Y.); 18977408221@163.com (R.L.); 15840260039@163.com (G.B.); lwx@fafu.edu.cn (W.L.); 2 Fujian Provincial Key Laboratory of Agroecological Processing and Safety Monitoring, Fujian Agriculture and Forestry University, Fuzhou 350002, China; 3 Key Laboratory of Crop Ecology and Molecular Physiology, Fujian Agriculture and Forestry University, Fuzhou 350002, China

**Keywords:** rice ratooning, yield, carbon footprint, rhizosphere microorganisms

## Abstract

Reducing greenhouse gas emissions while improving productivity is the core of sustainable agriculture development. In recent years, rice ratooning has developed rapidly in China and other Asian countries, becoming an effective measure to increase rice production and reduce greenhouse gas emissions in these regions. However, the lower yield of ratooning rice caused by the application of a single nitrogen fertilizer in the ratooning season has become one of the main reasons limiting the further development of rice ratooning. The combined application of nitrogen and phosphorus plays a crucial role in increasing crop yield and reducing greenhouse gas emissions. The effects of combined nitrogen and phosphorus application on ratooning rice remain unclear. Therefore, this paper aimed to investigate the effect of combined nitrogen and phosphorus application on ratooning rice. Two hybrid rice varieties, ‘Luyou 1831’ and ‘Yongyou 1540’, were used as experimental materials. A control treatment of nitrogen-only fertilization (187.50 kg·ha^−1^ N) was set, and six treatments were established by reducing nitrogen fertilizer by 10% (N1) and 20% (N2), and applying three levels of phosphorus fertilizer: N1P1 (168.75 kg·ha^−1^ N; 13.50 kg·ha^−1^ P), N1P2 (168.75 kg·ha^−1^ N; 27.00 kg·ha^−1^ P), N1P3 (168.75 kg·ha^−1^ N; 40.50 kg·ha^−1^ P), N2P1 (150.00 kg·ha^−1^ N; 13.50 kg·ha^−1^ P), N2P2 (150.00 kg·ha^−1^ N; 27.00 kg·ha^−1^ P), and N2P3 (150.00 kg·ha^−1^ N; 40.50 kg·ha^−1^ P). The effects of reduced nitrogen and increased phosphorus treatments in ratooning rice on the yield, the greenhouse gas emissions, and the community structure of rhizosphere soil microbes were examined. The results showed that the yield of ratooning rice in different treatments followed the sequence N1P2 > N1P1 > N1P3 > N2P3 > N2P2 > N2P1 > N. Specifically, under the N1P2 treatment, the average two-year yields of ‘Luyou 1831’ and ‘Yongyou 1540’ reached 8520.55 kg·ha^−1^ and 9184.90 kg·ha^−1^, respectively, representing increases of 74.30% and 25.79% compared to the N treatment. Different nitrogen and phosphorus application combinations also reduced methane emissions during the ratooning season. Appropriately combined nitrogen and phosphorus application reduced the relative contribution of stochastic processes in microbial community assembly, broadened the niche breadth of microbial communities, enhanced the abundance of functional genes related to methane-oxidizing bacteria and soil ammonia-oxidizing bacteria in the rhizosphere, and decreased the abundance of functional genes related to methanogenic and denitrifying bacteria, thereby reducing greenhouse gas emissions in the ratooning season. The carbon footprint of ratooning rice for ‘Luyou 1831’ and ‘Yongyou 1540’ decreased by 25.82% and 38.99%, respectively, under the N1P2 treatment compared to the N treatment. This study offered a new fertilization pattern for the green sustainable development of rice ratooning.

## 1. Introduction

Agriculture is widely regarded as a significant source of greenhouse gas emissions from human activities [1], with studies indicating that greenhouse gases (GHGs) emitted during rice production account for approximately 55% of global agricultural emissions [2], which is about four times the amount emitted by other cereals such as wheat or maize [3]. Rice ratooning involves the germination and growth of dormant buds on rice stubble after harvest to form panicles and then re-harvesting a rice crop [4]. Compared to double-cropping rice, rice ratooning not only has advantages such as shorter growth cycles and higher rice quality [5,6,7], but previous studies have also shown that per unit yield methane (CH_4_) emissions of rice ratooning are significantly lower compared to single and double-cropping rice. This reduction in global warming potential and greenhouse gas emission intensity contributes to the achievement of peak carbon and carbon neutrality in agriculture [8,9]. One of the important factors currently restricting the development of rice ratooning is its lower yield of the ratooning rice, which dampens farmers’ enthusiasm for planting. The use of a single type of fertilizer during the ratooning season is one of the factors contributing to lower yield, with nitrogen fertilizer being the main component of both bud-promoting and seedling-promoting fertilizers, despite its common use as a crop yield enhancer over the past few decades. However, the irrational application of nitrogen can increase greenhouse gas emissions, thereby affecting global climate change [10]. Studies by Wei et al. showed that the application of nitrogen fertilizer and nitrogen deposition could reduce soil respiration in forests [11]. On the other hand, research by Song et al. found that adding nitrogen fertilizer to wetland ecosystems significantly inhibits CH_4_ production, while the application of phosphorus has no direct effect [12]. However, combined application of N and P reduces CH_4_ production to a greater extent than the application of N alone. Studies by Lu et al. [13] and Zhu et al. [14] indicated that CH_4_ emissions from phosphorus-deficient soils decrease with increasing phosphorus fertilizer application, while other studies have suggested that the application of phosphorus alone can increase greenhouse gas emissions [15]. At the same time, previous studies have shown that the combined application of nitrogen and phosphorus fertilizers could significantly increase crop yield [16], and that an appropriate N:P ratio was key to maximizing crop yield, even more so than the absolute application amounts of nitrogen or phosphorus. The mechanisms by which nitrogen fertilizer affects greenhouse gas emissions are now fairly clear in rice, but there is still considerable debate over the impact of phosphorus fertilizers on methane emissions, and research on the combined application of nitrogen and phosphorus on greenhouse gas emissions in rice is still limited and mainly focused on single-season rice. The impact of combined nitrogen and phosphorus application in ratooning rice on yield and greenhouse gas emissions is still unclear.

Rhizosphere microorganisms plays a crucial role in driving material transformation in the soil, affecting not only plant nutrient use efficiency but also greenhouse gas emissions (GHG) [17,18,19]. In rice, the amount of CH_4_ emitted depends on the net balance between methanogenic bacteria and methane-oxidizing bacteria [20,21]. Methane-oxidizing bacteria, which are Gram-negative bacteria, use methane as their carbon source and energy, and are capable of oxidizing up to 90% of the CH_4_ produced by methanogens [22]. Nitrous oxide (N_2_O) emissions primarily depend on the transformations of nitrogen in soil nitrification and denitrification processes. Previous studies have shown that different fertilization treatments could affect the abundance of rhizosphere microorganisms related to greenhouse gas emissions in rice. The application of nitrogen fertilizer could increase the abundance of denitrifying bacteria in rhizosphere soil, which competed with methanogens for organic carbon. Additionally, denitrification produces toxic intermediates that inhibited methanogenic archaea in wetland sediments, thereby reducing CH_4_ production [22]. The application of phosphorus stimulated methanogenic activity, directly or indirectly increasing methanogen activity through fermentative bacteria that produced methanogenic substrates [23]. However, existing research on chemical fertilizers and rhizosphere microorganisms in rice ratooning has mainly focused on single chemical nitrogen fertilizers and the first season. There is still a lack of comprehensive and clear understanding of the impact of combined nitrogen and phosphorus fertilizer application on rhizosphere microbial ecology during ratooning season.

The development potential of rice ratooning is substantial, and under the premise of maintaining the current area of double-cropping rice, the planting area of rice ratooning in China can be increased by 11 times [24]. Therefore, optimizing fertilizer application during the ratooning season to increase the yield and reduce greenhouse gas emissions has become an urgent agricultural challenge. This study explored the impact of reduced nitrogen and increased phosphorus on ratooning rice yield, greenhouse gas emissions, and rhizosphere soil microorganisms, using two locally dominant rice varieties and different nitrogen and phosphorus application treatments during the ratooning season which would provide theoretical support for the energy-saving and green development of rice ratooning.

## 2. Results

### 2.1. Effects of Different Nitrogen and Phosphorus Fertilization Treatments in Ratooning Rice on the Yield

**Figure 1:**
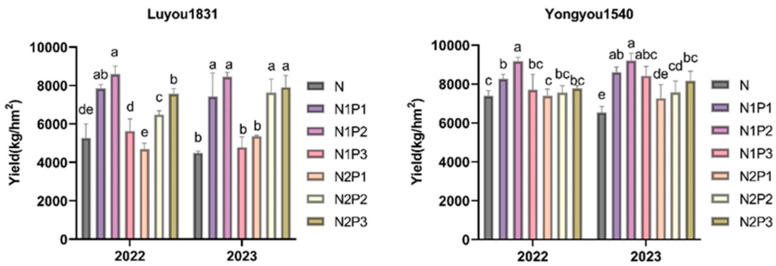
Changes in the yield of ratooning rice under various nitrogen and phosphorus fertilization treatments. Different letters in each column represent significant differences (*p* ≤ 0.05).

The ratooning crop yields of two consecutive years are shown in Figure 1. There were significant differences between the different nitrogen and phosphorus fertilizer treatments: N (187.50 kg·ha^−1^ N), N1P1 (168.75 kg·ha^−1^ N; 13.50 kg·ha^−1^ P), N1P2 (168.75 kg·ha^−1^ N; 27.00 kg·ha^−1^ P), N1P3 (168.75 kg·ha^−1^ N; 40.50 kg·ha^−1^ P), N2P1 (150.00 kg·ha^−1^ N; 13.50 kg·ha^−1^ P), N2P2 (150.00 kg·ha^−1^ N; 27.00 kg·ha^−1^ P), and N2P3 (150.00 kg·ha^−1^ N; 40.50 kg·ha^−1^ P), and a reduction in nitrogen coupled with an increase in phosphorus could improve yields. With a 10% reduction in nitrogen fertilizer, the yield of ratooning crop first increased and then decreased as the amount of phosphorus fertilizer increased. With a 20% reduction in nitrogen fertilizer, the yield showed an increasing trend as the amount of phosphorus fertilizer increased. For ‘Luyou 1831’, treatment N1P2 had the highest yield over two years, with an average yield of 8520.55 kg·ha^−1^, significantly higher than the N treatment (*p* < 0.05). The order of ratooning rice yield under different nitrogen and phosphorus fertilization treatments was N1P2 > N2P3 > N1P1 > N2P2 > N1P3 > N2P1 > N, where treatments N1P2, N2P3, N1P1, N2P2, N1P3, and N2P1 increased yields by 74.30%, 58.41%, 56.28%, 44.35%, 6.58%, and 2.67%, respectively, compared to the N treatment. For ‘Yongyou 1540’, treatment N1P2 also had the highest yield over two years, with an average yield of 9184.90 kg·ha^−1^, an increase of 25.79% over the N treatment. The order of ratooning crop yield under different nitrogen and phosphorus fertilization treatments was N1P2 > N1P1 > N1P3 > N2P3 > N2P2 > N2P1 > N, where treatments N1P1, N1P3, N2P3, N2P2, and N2P1 increased yields by 21.15%, 15.77%, 14.59%, 8.66%, and 5.33%, respectively, compared to the N treatment.

### 2.2. Effects of Different Nitrogen and Phosphorus Fertilization Treatments in Ratooning Rice on Greenhouse Gas Emissions

**Figure 2:**
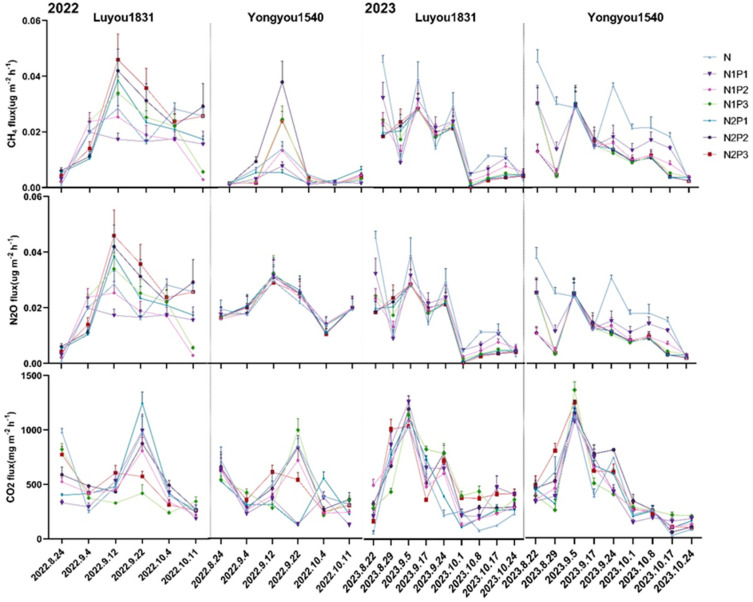
Dynamic changes of greenhouse gas emissions in ratooning rice under different nitrogen and phosphorus fertilization treatments.

**Figure 3:**
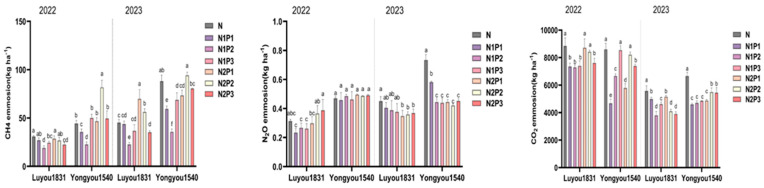
Changes in cumulative greenhouse gas emissions in ratooning rice under different nitrogen and phosphorus fertilization treatments. Different letters in each column represent significant differences (*p* ≤ 0.05).

During the ratooning season, methane (CH_4_) emissions showed a trend of initially increasing and then decreasing, with a peak around 25 days after the first season’s harvest (Figure 2). Significant differences in cumulative CH_4_ emissions were observed between treatments (Figure 3). With a 10% reduction in nitrogen fertilizer, both rice varieties showed a trend of initially decreasing and then increasing in cumulative methane emissions over the entire ratooning season as phosphorus fertilizer increased. However, with a 20% reduction in nitrogen fertilizer, ‘Luyou 1831’ showed a declining trend in cumulative methane emissions with increasing phosphorus fertilizer, while ‘Yongyou 1540’ exhibited a trend of initially increasing and then decreasing. Notably, under the N1P2 treatment, the cumulative methane emissions for both varieties were significantly reduced by 38.17% and 59.48%, respectively, compared to the N treatment.

Nitrous oxide (N_2_O) emissions during the ratooning season showed a trend of initially increasing and then decreasing, with a peak around 25 days after the first season’s harvest (Figure 2). With a 10% reduction in nitrogen fertilizer, ‘Luyou 1831’ showed an initial increase and then a decrease in cumulative N_2_O emissions in 2022, and a decreasing trend in 2023. ‘Yongyou 1540’ showed an increasing trend in cumulative N_2_O emissions in 2022, and a decreasing trend in 2023. With a 20% reduction in nitrogen fertilizer, ‘Luyou 1831’ showed an increasing trend in cumulative N_2_O emissions with increasing phosphorus fertilizer, whereas ‘Yongyou 1540’ showed an initial decrease followed by an increase. However, the differences in cumulative N_2_O emissions between treatments were inconsistent (Figure 3).

Carbon dioxide (CO_2_) emissions during the ratooning season typically exhibited an initial increase followed by a decrease, reaching a peak approximately 30 days after the first season’s harvest (Figure 2). With a 10% reduction in nitrogen fertilizer, ‘Luyou 1831’ showed an initial decrease and then an increase in cumulative CO_2_ emissions with increasing phosphorus fertilizer, while ‘Yongyou 1540’ showed a continuous increase. With a 20% reduction in nitrogen fertilizer, ‘Luyou 1831’ exhibited a declining trend in cumulative CO_2_ emissions as phosphorus fertilizer increased, while ‘Yongyou 1540’ showed an initial increase followed by a reduction. Notably, under the N1P1, N1P2, N2P3 treatments, both rice varieties displayed significantly diminished cumulative CO_2_ emissions compared to the conventional N treatment (Figure 3).

### 2.3. Effects of Different Nitrogen and Phosphorus Fertilization Treatments in Ratooning Rice on the Carbon Footprint

Significant differences were observed between global warming potential (GWP) and greenhouse gas intensity (GHGI) among the different nitrogen and phosphorus fertilization treatments in ratooning rice (Table 1). With a 10% reduction in nitrogen fertilizer application, both rice varieties exhibited an initial decrease followed by an increase in GWP and GHGI as phosphorus fertilizer application increased. Under N1P1 and N1P2 treatments, GWP and GHGI were lower than the N treatment. Specifically, under the N1P2 treatment, ‘Luyou 1831’ and ‘Yongyou 1540’ experienced significant decreases in GWP and GHGI by 41.94%, 67.49%, and 52.71%, 64.96%, respectively, compared to the N treatment. With a 20% reduction in nitrogen fertilizer application, ‘Luyou 1831’ demonstrated a decreasing trend in GWP and GHGI with increasing phosphorus fertilizer, with N2P3 treatment showing a significant decrease of 21.71% and 51.90% compared to the N treatment. On the other hand, ‘Yongyou 1540’ showed an initial increase followed by a decrease in GWP and GHGI with increased phosphorus fertilizer, with the two-year average for the N2P3 treatment showing decreases of 3.83% and 18.33% compared to the N treatment.

The carbon footprint of ratooning rice was calculated based on various production inputs. With a 10% reduction in nitrogen fertilizer application, the carbon footprint and yield-specific carbon footprint of the treatments that reduced nitrogen and increased phosphorus initially decrease but then increased with increasing phosphorus fertilizer application. Carbon efficiency initially increased but then decreased with increasing phosphorus application. Despite the increase in indirect emissions resulting from additional phosphorus application, the carbon footprint and yield-specific carbon footprint were lower than the N treatment due to reduced methane emissions, leading to higher carbon efficiency compared to the N treatment. When the nitrogen fertilizer application was reduced by 20%, the response of carbon footprint and carbon efficiency to phosphorus fertilizer varied between varieties. ‘Luyou 1831’ showed a decreasing trend in carbon footprint and yield-specific carbon footprint with increasing phosphorus application, while carbon efficiency showed the opposite trend. ‘Yongyou 1540’ exhibited an initial increase followed by a decrease in carbon footprint and yield-specific carbon footprint, whereas carbon efficiency showed an initial decrease followed by an increase. The carbon footprint for different treatments of ‘Luyou 1831’ followed the order of N2P1 > N2P2 > N > N1P1 > N1P3 > N2P3 > N1P2, with N1P1, N1P3, N2P3, and N1P2 treatments showing decreases of 5.51%, 10.15%, 13.37%, and 25.82% respectively, compared to the N treatment. For ‘Yongyou 1540’, the order was N2P2 > N > N2P3 > N1P3 > N2P1 > N1P1 > N1P2, with N2P3, N1P3, N2P1, N1P1, and N1P2 treatments showing decreases of 2.55%, 7.34%, 9.44%, 20.45%, and 38.99%, respectively, compared to the N treatment. Combining two years of data for both varieties, the carbon footprint under N1P1, N1P2, N1P3, N2P1, and N2P3 treatments decreased by 14.37%, 33.63%, 8.48%, 0.11%, and 6.95%, respectively, while N2P2 increased by 13.31% compared to the N treatment. The lowest carbon footprint for both varieties during the ratooning season was observed under the N1P2 treatment, with significant differences compared to the N treatment.

### 2.4. Effects of Appropriately Reduced Nitrogen and Increased Phosphorus Treatments in Ratooning Rice on Rhizosphere Soil Microbial Communities

**Figure 4:**
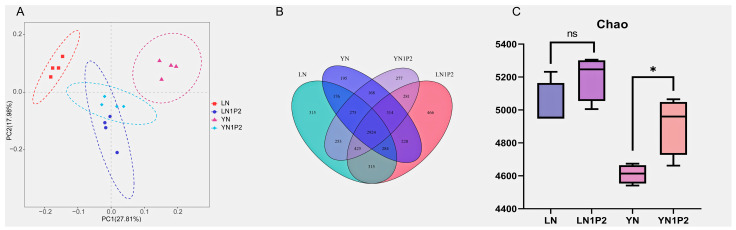
Principal component analysis (**A**), microbial diversity (**B**), and richness (**C**) assessments of rhizosphere soil microbial communities responding to appropriate nitrogen reduction and phosphorus increase treatments in ratooning rice. The ‘ns’ indicates a non-significant difference (*p* > 0.05). The symbols * indicate a significant difference (*p* < 0.05).

Principal component analysis (PCA) of rhizosphere microbes and microbial diversity assessments indicated that reduced nitrogen and increased phosphorus treatments in ratooning rice significantly influenced the composition of rhizosphere microbial communities, with some similarities discovered in the microbial communities of both varieties under reduced nitrogen and increased phosphorus treatments (Figure 4A). These treatments augmented the microbial diversity in the rhizosphere soil of ratooning rice (Figure 4B). Additionally, the richness of rhizosphere microbes was augmented by reduced nitrogen and increased phosphorus treatments (Figure 4C), with the Chao index showing a 3.56% escalation for ‘Luyou 1831’ under N1P2 treatment compared to the N treatment, and a significant 6.54% improvement for ‘Yongyou 1540’ under N1P2 treatment when contrasted with the N treatment.

### 2.5. Effects of Appropriately Reduced Nitrogen and Increased Phosphorus Treatments in Ratooning Rice on Rhizosphere Soil Microbial Community Assembly

**Figure 5:**
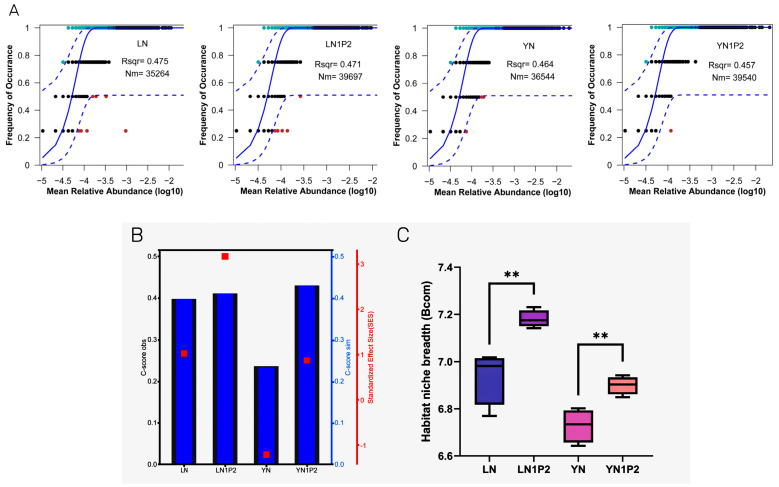
Analysis of neutral community model (**A**), checkerboard score (**B**), and niche width (**C**) in rhizosphere soil microbial communities responding to appropriate nitrogen reduction and phosphorus increase treatments in ratooning rice. The symbols ** indicates a highly significant difference (*p* < 0.01).

To gain a deeper understanding of how reduced nitrogen and increased phosphorus treatments affected microbial community structure, we integrated analyses using the neutral community model (NCM), checkerboard score (C-score), and niche width to explore the impact of reduced nitrogen and increased phosphorus on the assembly and construction processes of microbial communities during the ratooning season. Results from the NCM indicated that the relationship between the occurrence frequency of OTUs and their relative abundance could explain 47.5% and 46.4% of community variation under conventional nitrogen application (Figure 5A). In contrast, the N1P2 treatments could explain 47.1% and 45.7% of community variation, suggesting that the treatments reduced the relative contribution of stochastic processes in microbial community assembly. Zero-model analysis based on C-score (Figure 5B) revealed significant differences between the observed C-score and the simulated C-score across treatments, indicating non-random assembly patterns for rhizosphere microbial communities. Furthermore, the SES values for LN, LN1P2, and YN1P2 communities were positive, suggesting a segregated co-occurrence pattern of species within these communities. The SES value for the YN community was negative, indicating an aggregated co-occurrence pattern. The SES values for N1P2 treatments were greater than those for N treatments, indicating that reduced nitrogen and increased phosphorus treatments during the ratooning season enhanced the relative contribution of deterministic processes in microbial community assembly. By calculating the microbial niche width (Figure 5C), it was found that communities under N1P2 treatment had a significantly higher niche width than those under N treatment, suggesting that the appropriately reduced nitrogen and increased phosphorus treatments enhanced the rhizosphere microbial community’s resistance to external perturbations and their ability to utilize resources.

### 2.6. Effects of Appropriately Reduced Nitrogen and Increased Phosphorus in Ratooning Rice on Dominant Rhizosphere Soil Microbial Genera

**Figure 6:**
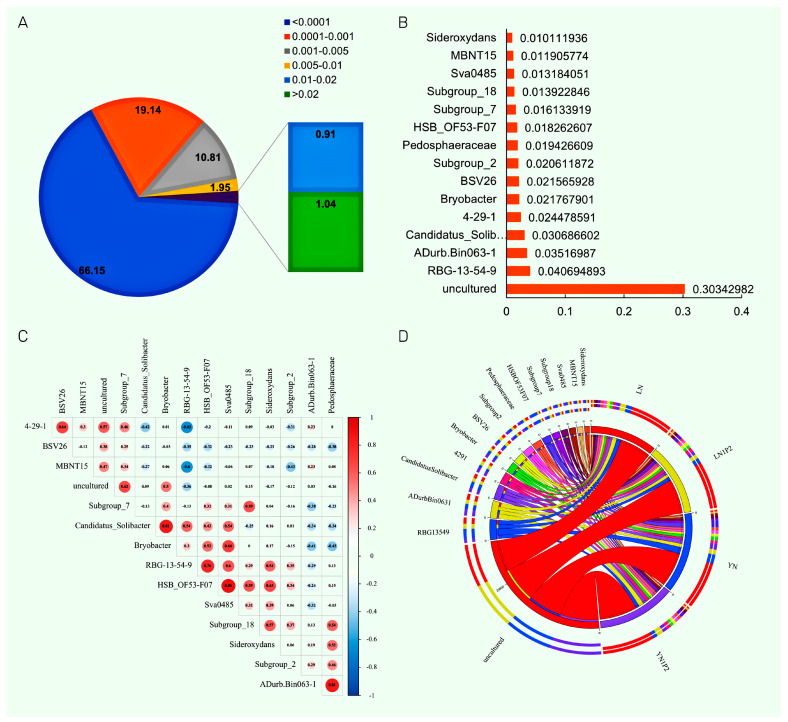
Dominant microbial genera (**A**), major dominant genera (**B**), correlation analysis of dominant genera (**C**), and abundance of dominant genera (**D**) in rhizosphere soil microbial communities responding to appropriate nitrogen reduction and phosphorus increase treatments in ratooning rice.

Dominant microbial genera were calculated at the bacterial genus level for all collected samples (Figure 6A) [25], with genera holding a dominance index >0.02 accounting for 1.04%, those with 0.01~0.02 making up 0.91%, those with 0.005~0.01 comprising 1.95%, those with 0.001~0.005 representing 10.81%, and those with 0.0001~0.001 constituting 19.14%. Using a dominance index threshold of >0.001, 15 major dominant genera were selected in the rhizosphere soil (Figure 6B), which included uncultured, *RBG-13-54-9*, *ADurb.Bin063-1*, *Candidatus_Solibacter*, *4-29-1*, *Bryobacter*, *BSV26*, *Subgroup_2*, *Pedosphaeraceae*, *HSB_OF53-F07*, *Subgroup_7*, *Subgroup_18*, *Sva0485*, *MBNT15*, and *Sideroxydans*. Among them, excluding unquantifiable genera, the most dominant genus was *RBG-13-54-9*, followed by *ADurb.Bin063-1* and *Candidatus_Solibacter*. Previous studies have shown that *ADurb.Bin063-1* [26], *Candidatus_Solibacter* [27], and *HSB_OF53-F07* [28] were bacteria involved in the decomposition of organic matter and utilization of carbon sources. Among these dominant genera, *BSV26* [29] and *Sva0485* [30] were associated with nitrification and denitrification processes in the soil, while *4-29-1* [31] was positively correlated with soil phosphatase activity. Thus, the main dominant genera in the rice rhizosphere soil played certain roles in the soil elements C, N, and P cycles, which was beneficial for investigating the impact of microbial communities on the carbon footprint of rice fields.

Correlation analysis of dominant genera (Figure 6C) revealed a positive association between *HSB_OF53-F07* and *Candidatus_Solibacter*, two genera related to carbon source decomposition, and they also correlated with *Sva0485*, a genus associated with soil nitrifying archaea. *ADurb.Bin063-1* showed a positive correlation with genera involved in the soil phosphorus cycle and iron oxidation. Further investigation into the impact of nitrogen and phosphorus fertilization on dominant rhizosphere genera in ratooning rice (Figure 6D) indicated that N1P2 treatments decreased the abundance of genera such as *RBG-13-54-9*, *Bryobacter*, *HSB_OF53-F07*, *Sva0485*, and *Sideroxydans*. On the other hand, N1P2 treatment increased the abundance of *ADurb.Bin063-1*, *4-29-1*, *BSV26*, and *Pedosphaeraceae*. Overall, appropriately reduced nitrogen and increased phosphorus treatments enhanced the abundance of genera related to soil phosphorus cycling and ammonification, while decreasing the abundance of those involved in nitrification.

### 2.7. Prediction of Rhizosphere Soil Microbial Community Functional Genes

**Figure 7:**
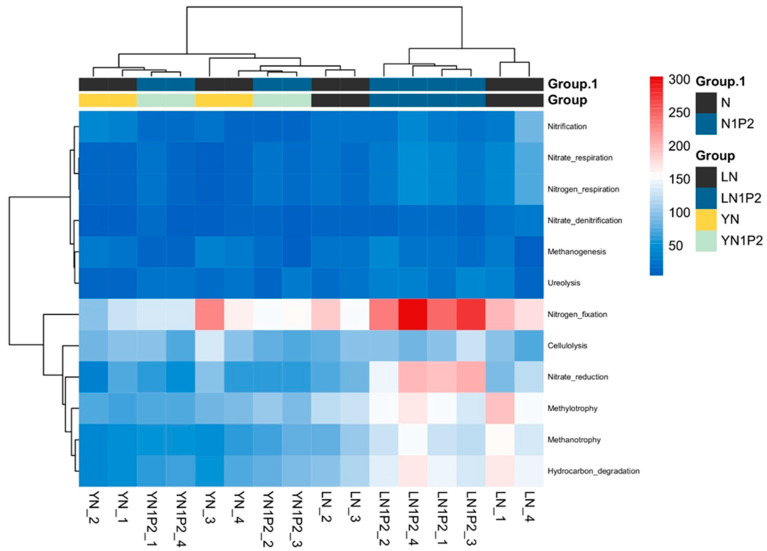
Effects of appropriately reduced nitrogen and increased phosphorus treatments in ratooning rice on the functional aspects of the rhizosphere soil microbial community.

The FAPROTAX functional gene prediction tool was used to compare existing 16S rRNA gene sequencing data with a reference genome database of microbes with known metabolic functions. Consequently, 71 gene families were predicted in the rhizosphere soil samples of ratoon rice. From these, 12 gene families related to soil nitrogen cycling, carbon source transformation, and methane emissions were selected for further analysis (Figure 7). Analysis revealed that N1P2 treatments increased the gene abundance of microbial genera associated with methane oxidation, methylotrophy, nitrogen fixation, hydrocarbon degradation (organic matter decomposition), and urea decomposition; and decreased the gene abundance of methanogenic genera and those related to nitrification and nitrate denitrification. The appropriately reduced nitrogen and increased phosphorus treatments increased the abundance of methane-oxidizing bacterial genera and reduced the gene abundance of methanogenic as well as nitrifying and denitrifying bacterial genera, which was consistent with the greenhouse gas emissions data collected earlier in the study.

## 3. Discussion

### 3.1. Effects of Different Nitrogen and Phosphorus Fertilization Treatments in Ratooning Rice on Yield and Greenhouse Gas Emissions

Nitrogen and phosphorus are two of the three essential nutrients for plant growth and development. There are certain interactions between these two elements that can significantly affect crop yields. Dai et al. [32] found, through a 16-year fixed-point trial, that the combined application of nitrogen and phosphorus significantly increased the yields of wheat and corn compared to the application of nitrogen or phosphorus alone or the combination of nitrogen and potassium. Research by Elke et al. [33] suggested that adding trace phosphorus could enhance the growth and development of rice seedlings, leading to higher yield. The result of this study showed that within a certain range of nitrogen reduction, nitrogen and phosphorus fertilization treatments during the ratooning season could significantly increase the yield of ratooning rice compared to the nitrogen-only treatments. This suggests that nitrogen–phosphorus interactions could mitigate the adverse effects caused by reduced nitrogen and increased yields. With a 10% reduction in nitrogen fertilizer (applying 168.75 kg·ha^−1^ N), the yield of ratooning rice initially increased and then decreased with the increase in phosphorus fertilizer application. When the nitrogen fertilizer application was sufficient, applying a certain amount of phosphorus fertilizer could promote the growth and development of ratooning rice and increase the yield. However, exceeding a certain amount could suppress the yield of ratooning rice. With a 20% reduction in nitrogen fertilizer (applying 150.00 kg·ha^−1^ N), the yield of ratooning rice showed an increasing trend with the increase in phosphorus application. This may be due to the nutrient deficiency caused by the 20% reduction in nitrogen fertilizer. The addition of phosphorus could compensate for this nutrient deficiency, mitigating the adverse effect on ratooning rice yield caused by reduced nitrogen. Different types of chemical fertilizers and application ratios not only affected crop yield formation but also greenhouse gas emissions. Shen et al. [4] conducted a comprehensive analysis of 561 rice ratooning planting farms in Hubei Province, revealing that chemical fertilizers, mechanization of field operations, and water management were the main environmental factors influencing methane emissions in the rice ratooning system. The result of this study indicated that, compared to traditional nitrogen-only applications, reduced nitrogen and increased phosphorus during the ratooning season could significantly reduce CH_4_ and CO_2_ emissions, while differences in N_2_O emissions between different treatments were not significant. Kim et al. [12] believed that adding nitrogen fertilizer to soil helped reduce methane emissions, while the addition of phosphorus had no significant impact on greenhouse emissions. This study showed that with a 10% reduction in nitrogen fertilizer, the cumulative methane emissions first decreased and then increased with more phosphorus application. With a 20% reduction in nitrogen, a different trend was observed, which may be due to the different nitrogen to phosphorus ratios affecting the efficiency of carbon and nitrogen transformation in the rice fields. Previous research has shown that a low nitrogen–phosphorus ratio could reduce soil nitrification, and nitrification could affect methane emissions from rice fields, which was consistent with our results.

Converting methane and nitrous oxide emissions into carbon dioxide equivalents, we calculated the impact of different treatments on global warming potential (GWP) and carbon emission intensity of greenhouse gas emissions during the ratooning season. The result indicated that the N1P2 treatment could significantly reduce both the global warming potential and greenhouse gas emission intensity of ratooning rice. This was likely due to the inhibitory effect of nitrogen–phosphorus interactions on methane emissions and the fact that chemical fertilizers were applied only once during the ratooning season, which facilitated rice absorption of nitrogen, reduced nitrogen loss, and consequently lowered greenhouse gas emissions. Similar findings were reported by Wei et al. [34] in subtropical forest soils, where combined nitrogen and phosphorus fertilization could significantly suppress greenhouse gas emissions. The calculation of the carbon footprint allowed a comprehensive understanding of the direct and indirect greenhouse gas emissions during the rice production process, thus enabling accurate assessment of the impact of different rice cultivation methods on the ecological environment [35]. The carbon footprint of different crops varies significantly due to factors such as yield, agricultural input investment, and field management practices [36]. Wang et al. [37] reported that the carbon footprint of different fertilizer types ranks as follows: phosphorus > nitrogen > potassium. In this study, although increasing phosphorus fertilization under the treatment of reducing nitrogen by 10% raised indirect emissions of ratooning rice, the overall carbon footprint was reduced. This was likely because, while the addition of phosphorus fertilizer increased its indirect emissions, the combined application of nitrogen and phosphorus significantly suppressed methane emissions, offsetting the adverse environmental impact of increased phosphorus application, thereby affecting the carbon footprint of ratooning rice. Therefore, optimizing the type of fertilization and selecting an appropriate fertilization ratio was of great significance for ensuring rice production safety and developing conservation-oriented, environmentally friendly agriculture [38].

### 3.2. Effects of Different Nitrogen and Phosphorus Fertilization Treatments in Ratooning Rice on Rhizosphere Microbial Communities

The diversity and biomass of soil microbial communities play a crucial role in regulating fundamental ecosystem processes such as organic matter decomposition, nutrient cycling, and gas flux [39,40]. The rhizosphere microbiome, a crucial component of the soil microbiome, is closely related to plant growth and health, hence often referred to as the “second genome” of the plant [41,42]. The structure of the rhizosphere microbial community is dynamic during plant development and is significantly associated with the type of fertilization. Differing microbial species and activity are observed under various fertilizer types. Research has shown that long-term nitrogen fertilization could lead to soil acidification, reducing the abundance and diversity of soil microbial communities [43]. Dong et al. [41] also found that increasing nitrogen application had a negative impact on soil microbial community structure. Our study, through principal component analysis and microbial diversity assessment, revealed significant differences in the composition of rhizosphere microbial communities of ratooning rice between the appropriately reduced nitrogen and increased phosphorus treatment and the conventional nitrogen-only treatment, with the former increasing the abundance and diversity of rhizosphere microbes. This may be due to the reduction in nitrogen fertilizer application and the increase in phosphorus affecting the variation of nutrient content in the soil, thereby promoting microbial enrichment. It could also be due to the combined application of nitrogen and phosphorus fertilizers enhancing plant nitrogen uptake and soil carbon transformation, improving the carbon source required for microbial growth in the rhizosphere, promoting the secretion of root exudates in ratoon rice, and thereby recruiting different microbial genera. The result of this study indicated that the combined application of nitrogen and phosphorus fertilizers enhanced the abundance of methanotrophic and ammonia-oxidizing bacteria in the soil, while decreasing the abundance of methanogenic bacteria and those involved in nitrification and denitrification processes. Consequently, the combined application of nitrogen and phosphorus fertilizers reduced greenhouse gas emissions by affecting changes in the microbial community, which was generally consistent with the research conducted by Kim et al. [12]. Larmola et al. [44] found that phosphorus could increase nitrogen fixation in silt soil and the abundance of methanotrophic bacteria, which was aligned with our experimental results. Zhang et al. [45] and Conrad et al. [46] also demonstrated that phosphorus fertilization could stimulate soil methane uptake and inhibit aceticlastic methanogenesis activity in the rice rhizosphere, thereby enhancing the activity of methanotrophic bacteria and reducing soil methane emissions. However, the molecular mechanisms by which the combined addition of nitrogen and phosphorus affected these bacterial genera were still not entirely clear, and the impact of different nitrogen-to-phosphorus ratios on microbial communities requires further in-depth investigation.

## 4. Materials and Methods

### 4.1. Experimental Site Overview

The experiment was conducted in 2022 and 2023 at the Rice Ratooning Science and Technology Courtyard in Shipotown, Pucheng County, Nanping City, Fujian Province (118°35′ E, 27°68′ N). The experimental site is used for rice cultivation year-round, with a mid-subtropical monsoon climate, distinct seasons, and abundant rainfall. The soil type of the experimental area is sandy loam, with a soil pH of 6.18, total nitrogen 1.25 g·kg^−2^, total phosphorus 0.35 g·kg^−2^, total potassium 5.24 g·kg^−2^, nitrate nitrogen 34.60 mg·kg^−2^, ammonium nitrogen 64.95 mg·kg^−2^, available phosphorus 11.76 mg·kg^−2^, and organic matter 16.90 mg·kg^−2^.

### 4.2. Experimental Design

The experiment used hybrid rice ‘Luyou 1831’ and ‘Yongyou 1540’ as test materials, with a completely randomized block design. Fertilization treatments during the ratooning season were based on a nitrogen application of 187.50 kg·ha^−1^, with a total nitrogen application reduced by 10% (168.75 kg·ha^−1^) and by 20% (150.00 kg·ha^−1^). On the basis of reduced nitrogen, three levels of phosphorus fertilizers (13.50 kg·ha^−1^, 27.00 kg·ha^−1^, 40.50 kg·ha^−1^) were applied, constituting six treatments: N1P1 (168.75 kg·ha^−1^ N; 13.50 kg·ha^−1^ P), N1P2 (168.75 kg·ha^−1^ N; 27.00 kg·ha^−1^ P), N1P3 (168.75 kg·ha^−1^ N; 40.50 kg·ha^−1^ P), N2P1 (150.00 kg·ha^−1^ N; 13.50 kg·ha^−1^ P), N2P2 (150.00 kg·ha^−1^ N; 27.00 kg·ha^−1^ P), and N2P3 (150.00 kg·ha^−1^ N; 40.50 kg·ha^−1^ P). The control was conventional nitrogen-only fertilization (187.50 kg·ha^−1^ N). There were two varieties, with three replications each, totaling 42 plots. Each experimental plot was 15 m × 20 m, separated by ridges wrapped in plastic film, and surrounded by drainage ditches to ensure independent irrigation and drainage. In 2022, rice ratooning seedlings were cultivated on March 14, transplanted on April 19, with a plant spacing of 30 cm × 17 cm, harvested on August 13 for the first season, headed on September 20 for the ratooning season, and harvested on October 23. In 2023, rice ratooning seedlings were cultivated on March 6, transplanted on April 11, with the same plant spacing, harvested on August 15 for the first season, headed on September 17 for the ratooning season, and harvested on October 26.

Fertilization for the first season followed conventional high-yield cultivation management, applying pure nitrogen 225.00 kg·ha^−1^, pure phosphorus 67.50 kg·ha^−1^, and pure potassium 180.00 kg·ha^−1^. The fertilizers used were 46% urea, 12% calcium superphosphate, and 60% potassium chloride produced by Shaanxi Shaanhua Coal Chemical Group Co., Ltd. (Weinan, China). The amount of fertilizer used for bud-promoting was not included in the first season’s nitrogen application. The fertilization amount for the ratooning season was the total of bud-promoting and seedling-promoting fertilizers, with a 5:5 ratio between them. Bud-promoting fertilizer was applied 20 days after the first season heading, and seedling-promoting fertilizer was applied 5 days after the first season harvest. When the tillers in the first season reached 80% of the expected panicle number, one mid-season drainage was conducted, and another drainage was implemented during the late grain-filling stage of the first season; for the rest of the period, the field was managed according to conventional high-yield cultivation practices, with a stubble height of 25 cm left for the ratooning crop.

### 4.3. Measurement Items and Methods

#### 4.3.1. Field Growth Meteorological Data Measurement

**Figure 8:**
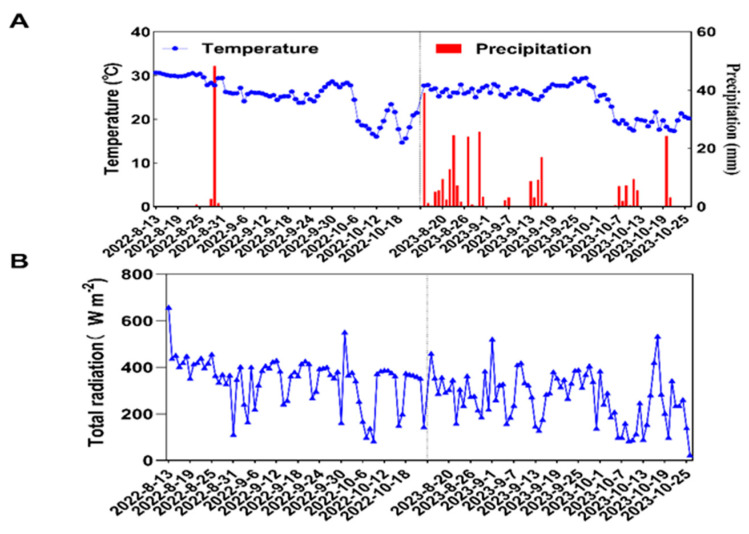
Daily average rainfall, average temperature, and total radiation in ratooning rice. (**A**) Average daily rainfall and temperature in 2022 and 2023; (**B**) Total radiation in 2022 and 2023.

The daily average rainfall, average temperature, and total radiation during the ratooning season in 2022 and 2023 are shown in Figure 8. The daily average temperature during the ratooning season of 2022 was 25.28 °C, with total rainfall of 53.20 mm, and the daily average total radiation was 346.67 W/m^2^. In the ratooning season of 2023, the daily average temperature was 24.50 °C, total rainfall was 270.91 mm, and the daily average total radiation was 269.17 W·m^−2^.

#### 4.3.2. Yield Measurement of the Ratooning Crop

During the maturity period of the ratooning crop, 3 representative field blocks were randomly selected from each treatment for manual harvesting to measure the yield. The actual harvested area was 2 m × 2 m per block, and the rice was sun-dried and threshed to determine the yield after removing impurities.

#### 4.3.3. Collection and Measurement of Greenhouse Gases

Gas collection was conducted using a closed static dark box observation method. The sampling box, made of PMMA material, was a cylindrical container with an inner diameter of 30 cm and a height of 100 cm. The external cylinder and top cover of the box were covered with tin foil stickers for light shielding and heat insulation. The top cover was removable and equipped with a small fan and a hanging temperature probe for gas mixing and internal temperature monitoring. Gas collection started 8 days after the harvest of the previous season and continued until the end of the regrowth season’s maturation stage. Gas samples were collected at intervals of 7–10 days. The sampling time was set from 9:00 AM to 11:00 AM. A 15 cm high flanged circular base was embedded into the soil one day before gas collection. Prior to sampling, the flanged cylinder and base were secured with nuts and sealed with rubber rings. The water level distance from the top cover was measured, and then the top cover was fixed to begin gas collection. A total of three collections were made, with a time interval of 10 min for each collection, and 200 mL of gas was extracted and the temperature inside the cylinder was recorded.

Gas chromatography (Agilent 7890B, Agilent Technologies Inc., Wilmington, DC, USA) was used to detect gas concentrations. CH_4_ and CO_2_ gas concentrations were detected automatically by a Flame Ionization Detector (FID), and N_2_O gas concentration by an electron capture detector (ECD). The operating temperature was 200 °C, with a chromatographic column temperature of 55 °C. Standard gases were provided by Foshan Kede Gas Chemical Industry Co., Ltd. (Foshan, China).

The calculation equation for soil CO_2_ emission flux is:

$$F=\frac{12}{22.41}\times \frac{\Delta C}{\Delta t}\times \frac{V}{S}\times \frac{273}{\left(273+T\right)}\times 1000\times 600$$

where F is the CO_2_ emission flux (mg·C·m^−2^·h^−1^); 22.41 is the molar volume of CO_2_ at standard conditions (L·mol^−1^); 12 is the molar mass of carbon (g·mol^−1^); V is the effective volume inside the sampling chamber (L); S is the soil area covered by the sampling chamber (m^2^); ΔC is the gas concentration difference (µL^−1^); Δt is the sampling interval (min); T is the temperature inside the sampling chamber (°C); 1000 converts g to mg; 60 converts minutes (min) to hours (h).

The calculation equation for soil N_2_O (CH_4_) emission flux is:

$$F=\rho \times \frac{V}{A}\times 100\times P/P{}^{\circ}\times \frac{273}{273+T}\times \frac{\Delta C}{\Delta t}\times 60$$

where F is the N_2_O (CH_4_) emission flux (µg N_2_O­N m ^−2^ h^−1^); ρ is the density of N_2_O (CH_4_) at standard conditions (µg N_2_O-N m^−3^); V represents the volume of the enclosed static chamber (cm^3^); A is the surface area of the soil inside the sampling chamber (cm^2^); P is the pressure inside the enclosed static chamber (Pa); P0 is the atmospheric pressure at standard conditions, 1.013 × 10⁵ Pa, the atmospheric pressure in the experimental area is roughly equivalent to that at standard conditions, thus P/P0 is approximately 1; T is the temperature inside the enclosed static chamber (°C). ΔC represents the change in N_2_O concentration within the enclosed static chamber per unit of time (10^−^⁹ min^−1^), which is the slope of the linear regression curve between N_2_O concentration and time; if R^2^ is less than 0.9, the regression coefficient is not valid; Δt is the sampling interval (min); 60 converts minutes (min) to hours (h).

The calculation equation for the total greenhouse gas emission is:

$$f\equiv \sum_{i=1}^{n}\left[(F_{i}+F_{i-1})/2\times D\times 24\times 10^{-2}\right]$$

where f is the greenhouse gas emission (kg·ha^−1^), n is the total number of greenhouse gas collections, F_i_ and F_i−1_ are the flux values F (mg·m^−2^·h^−1^) of the greenhouse gases measured at the i-th and (i−1)-th times, respectively, and D is the interval in days (d) between two consecutive collections.

#### 4.3.4. Calculation of Global Warming Potential and Greenhouse Gas Emission Intensity

Global warming potential (GWP) is commonly used to assess the ability of various greenhouse gases to create a greenhouse effect corresponding to the same effect as CO_2_ equivalents [47]. On a 100-year time scale, the warming potential values for CH_4_ and N_2_O are 25 and 298, respectively [48]. In calculating the total CO_2_ equivalents, the CO_2_ emissions from rice fields are not included. Therefore, the comprehensive assessment equation for greenhouse gas emissions from rice fields based on GWP is:

$$GWP=25\times f\left(CH_{4}\right)+298\times f(N_{2}O)$$

where GWP is the global warming potential (kg CO_2_-eq·ha^−1^), f(CH_4_) and f(N_2_O) are the total emissions of CH_4_ and N_2_O from the rice fields, respectively.

Greenhouse gas emission intensity (GHGI) is the ratio of the total warming potential of CH_4_ and N_2_O to the crop yield; it is an index that comprehensively evaluates the greenhouse effects of each treatment [49]. The calculation equation is:

GHGI=GWP/Y

where GHGI is the greenhouse gas emission intensity (kg CO_2_-eq·t^−1^), and Y is the total rice yield (t·ha^−1^).

#### 4.3.5. Carbon Footprint Study Boundary and Estimation Method

The carbon footprint (CF) is based on the production input during the entire growing period of rice from sowing to harvest and is the sum of all direct and indirect greenhouse gas emissions expressed in carbon dioxide equivalents (CO_2_-eq) [50]. Greenhouse gas emissions within the life cycle mainly include those from the indirect fossil fuel consumption for agricultural input and management, and the total emissions of greenhouse gases produced by rice field soils. Therefore, this study defines the boundaries for accounting for greenhouse gas emissions from the ratoon rice production system as: (1) greenhouse gas emissions generated during the transportation and consumption of nitrogen and phosphorus fertilizers; (2) greenhouse gases emitted from the indirect consumption of fossil fuels for diesel fuel used in paddy cultivation and harvesting machinery and electricity for irrigation; (3) non-CO_2_ emissions from rice fields during growth, mainly including CH_4_ and N_2_O emissions (as rice has the ability to photosynthetically fix CO_2_, the net emission flux of CO_2_ is actually negative, hence it is not included in the statistics).

The carbon footprint estimation equation is:

$$CFA=\sum_{i}^{n}({Al}_{i}\times EF_{i})+E\left(CH_{4}\right)+E(N_{2}O)$$

Here, CFA is the total carbon footprint in rice production (kg CO_2_-eq·ha^−1^); ${Al}_{i}$ is the amount of a certain agricultural input (kg), such as seeds, fertilizers, agricultural film, pesticides, electricity for irrigation, and fuel for cultivation and harvesting, representing the emission factor for greenhouse gases formed by the indirect consumption of fossil fuels (kg CO_2_-eq·kg^−1^); E(CH_4_) and E(N_2_O) are the cumulative emissions of CH_4_ and N_2_O from rice fields converted to CO_2_ equivalents (kg CO_2_-eq·ha^−1^).

The carbon footprint per unit yield calculation equation:

CFy=CFA/Y

The carbon efficiency (CE) calculation equation:

CE=Y/CFA

Here, CFy is the carbon footprint per unit yield (kg CO_2_-eq·t^−1^); CE is carbon efficiency (t·kg^−1^ CO_2_-eq); CFA is the total carbon footprint in rice production (kg CO_2_-eq·ha^−1^); Y is the total rice yield (t·ha^−1^).

#### 4.3.6. Rhizosphere Soil Sample Collection

Fifteen representative rice plants from each variety, exhibiting uniform growth characteristics, were selected from plots subjected to both optimal treatment and conventional nitrogen treatment. The complete root system of the rice plants was excavated, and the soil surrounding the root zone was removed using the shaking method. After that, the soil attached to the roots within 5 mm was gently shaken into self-sealed bags, and the soil samples were mixed and stored at low temperature.

#### 4.3.7. Extraction and PCR Amplification of Total DNA from Rhizosphere Soil Microorganisms

Root-associated soil microbial DNA extraction: Root-associated soil microbial DNA was extracted using the BioFast Soil Genomic DNA Extraction Kit, produced by Hangzhou Borui Technology Co., Ltd. (Hangzhou, China). A total of 100 μL elution buffer was used for elution. The extracted genomic DNA was then analyzed using 1% agarose gel electrophoresis. The quality of the extracted DNA samples was assessed using spectrophotometry (260 nm/280 nm absorbance ratio). After the analysis, the samples were stored at −20 °C for future experimental use. For microbial diversity analysis, the bacterial 16S rRNA V3~V4 region was targeted. The DNA samples were sent to Bejing Allwegene Technology Co., Ltd. (Bejing, China) for sequencing using the Illumina Miseq PE300 high-throughput sequencing platform. The specific primers used for amplifying the bacterial 16S rRNA V3~V4 region were 338F (5′-ACTCCTACGGGAGGCAGCAG-3′) and 806R (5′-GGACTACNNGGGTATCTAAT-3′). The PCR reaction mixture included 12.5 μL 2×Taq PCR MasterMix, 3 μL BSA (2 ng/μL), 2 μL each primer (5 μmol/L), 2 μL template DNA, and 5.5 μL ddH_2_O. The reaction program consisted of an initial denaturation at 95 °C for 5 min; followed by 32 cycles of denaturation at 95 °C for 45 s, annealing at 55 °C for 50 s, and extension at 72 °C for 45 s; and a final extension at 72 °C for 10 min. The raw sequencing data were uploaded to the NCBI SRA database.

### 4.4. Data Processing and Analysis

We performed statistical analysis using IBM SPSS version 26 software and created graphs using Excel 2016 and GraphPad Prism 9.5.

## 5. Conclusions

Compared with conventional nitrogen fertilization, appropriate reduction of nitrogen and increase of phosphorus during the ratooning season significantly improved yield and affected the structure of rhizosphere soil microbial communities in ratooning rice. The appropriate nitrogen reduction and phosphorus increase during the ratooning season influenced the determinism in the microbial community assembly process in the rhizosphere soil, enhancing the niche breadth and microbial abundance, thus providing a healthier rhizosphere environment for high-yielding ratoon rice. The treatment increased the abundance of methanotrophic bacteria and those related to soil ammonia oxidation while decreasing the abundance of methanogenic bacteria and those related to soil nitrification and denitrification, thereby inhibiting methane production and greenhouse gas emissions. This led to reduced carbon emissions and improved carbon efficiency in ratooning rice. In summary, the recommended optimal nitrogen and phosphorus fertilizer ratio for ratooning rice was N1P2, which reduced the carbon footprint, increased the yield, and improved the carbon efficiency of the production process, offering a new fertilization pattern for the green sustainable development of rice ratooning.

## Figures and Tables

**Table 1:** Changes in indirect emissions, GWP, GHGI, carbon footprint per area (CFA), carbon footprint per yield (CFy), and carbon efficiency (CE) in ratooning rice under different nitrogen and phosphorus fertilization treatments.

Year	Varieties	Treatments	Indirect Emissions (kg CO_2_-eq·ha^−1^)	GWP (kg CO_2_-eq·ha^−1^)	GHGI (kg CO_2_-eq·ha^−1^)	CFA (kg CO_2_-eq·ha^−1^)	CFy (kg CO_2_-eq·ha^−1^)	CE (t·kg^−1^ CO_2_-eq)
2022	Luyou 1831	N	608.07	867.55 a	166.37 a	1475.63 a	0.28 a	3.57 d
		N1P1	601.39	748.95 bc	95.32 c	1350.34 b	0.17 c	5.82 b
		N1P2	623.40	558.38 d	64.96 d	1181.78 c	0.14 c	7.27 a
		N1P3	644.59	689.87 c	123.22 b	1334.45 b	0.24 b	4.21 c
		N2P1	572.70	808.93 ab	173.52 a	1381.63 ab	0.3 a	3.4 d
		N2P2	594.71	778.77 abc	120.02 b	1373.48 ab	0.21 b	4.73 c
		N2P3	615.90	678.9 c	89.54 cd	1294.8 b	0.17 c	5.86 b
	Yongyou 1540	N	608.07	1253.38 b	169.62 b	1861.45 b	0.25 b	3.97 c
		N1P1	601.39	1029.41 c	124.76 c	1630.8 c	0.2 c	5.08 b
		N1P2	623.40	713.24 d	77.76 d	1336.64 d	0.15 d	6.86 a
		N1P3	644.59	1391.78 b	182.5 b	2036.37 b	0.27 b	3.8 c
		N2P1	572.70	1317.74 b	178.09 b	1890.44 b	0.26 b	3.92 c
		N2P2	594.71	2190.03 a	290.97 a	2784.74 a	0.37 a	2.73 d
		N2P3	615.90	1388.6 b	178.44 b	2004.5 b	0.26 b	3.91 c
2023	Luyou 1831	N	608.07	1271.1 c	282.59 b	1879.17 c	0.42 ab	2.4 e
		N1P1	601.39	1218.09 cd	168.55 cd	1819.49 cd	0.25 cd	4.1 c
		N1P2	623.40	683.15 e	80.97 e	1306.55 e	0.15 e	6.48 a
		N1P3	644.59	1035.17 d	219.31 c	1679.75 cd	0.36 b	2.89 de
		N2P1	572.70	1853.6 a	346.24 a	2426.3 a	0.45 a	2.22 e
		N2P2	594.71	1517.33 b	199.47 c	2112.04 b	0.28 c	3.61 cd
		N2P3	615.90	995.34 d	126.39 de	1611.24 d	0.2 de	4.91 b
	Yongyou 1540	N	608.07	2425.24 a	371.38 a	3033.32 a	0.46 a	2.16 e
		N1P1	601.39	1661.31 d	193.03 d	2262.7 d	0.26 e	3.81 b
		N1P2	623.40	1026.26 e	111.76 e	1649.66 e	0.18 f	5.59 a
		N1P3	644.59	1854.07 cd	221.29 cd	2498.66 c	0.3 de	3.39 bc
		N2P1	572.70	1969.23 bc	273.11 b	2541.93 c	0.35 c	2.87 cd
		N2P2	594.71	2482.32 a	328.68 a	3077.03 a	0.41 b	2.46 de
		N2P3	615.90	2149.13 b	263.4 bc	2765.03 b	0.34 cd	2.96 cd

Note: Different letters in each column of the same rice variety represent significant differences (*p* ≤ 0.05).

## Data Availability

Data are contained within the article, further inquiries can be directed to the corresponding author.
